# Adherence to Lifestyle Recommendations among Adults Attending Hypertension Clinics in Selected Hospitals in Tanzania: A Cross-Sectional Study

**DOI:** 10.24248/eahrj.v8i1.748

**Published:** 2024-03-28

**Authors:** Joseph Nyanda Shilole, Rehema Bakari Omari, Jacktan Josephat Ruhighira, Ahmed Gharib Khamis, Julius Edward Ntwenya

**Affiliations:** a Department of Public Health, The University of Dodoma, Tanzania; b Department of Epidemiology and Biostatistics; Muhimbili University of Health and Allied Sciences, Tanzania; c Department of Clinical Nursing, The University of Dodoma, Tanzania

## Abstract

**Background and Aims:**

Hypertension is the first contributor to the deaths caused by non-communicable diseases (NCDs) worldwide. A change of lifestyle is recommended as an equal-first-line approach for controlling hypertension. However, the burden of uncontrolled hypertension remains high. This article describes the level of adherence to recommended lifestyle modifications among hypertensive patients in Tanzania.

**Methods:**

The research was carried out from June to September 2020 using a cross-sectional study that involved an interviewer-administered questionnaire with 311 participants. These were patients with hypertension (> 18 years old) who were randomly selected from patients attending clinics during the study period. The lifestyle behaviours were assessed using the WHO Steps survey standard questionnaire. SPSS, version 26, was used to enter and analyse the data.

**Results:**

The mean age of hypertensive patients was 53.6 ± 7.5 years. Females were 58.8%. Only 17.7% had good compliance with the recommended lifestyle behaviours related to hypertension. Regular physical activities had 37.9% adherence, 99% adhered to non-smoking, 94.2% adhered to moderation of alcohol consumption, and 22.2% adhered to the consumption of fruits and vegetables. Patients with adequate knowledge were two times more likely to comply with the WHO recommended lifestyle behaviours (aOR=2.32; 95% confidence interval [CI], 1.082 to 3.471; *P= .05*]

**Conclusion:**

Most patients with hypertension had poor lifestyle behaviours for the management of hypertension, with varying level of adherence to the recommended life style changes.

## BACKGROUND

Hypertension is the leading global health problem.^1^ More than 1 billion adults (22%) in the world were estimated to be hypertensive.^2^ The number was expected to increase to 1.5 billion by 2025.^3^ The prevalence is highest in low- and middle-income countries (LMICs), mostly (27%) affecting the African regions.^4^ Consequently, hypertension is a chief contributor to the deaths from noncommunicable diseases (NCDs) worldwide.^2^ A high burden of uncontrolled hypertension is reported in Tanzania.^5^ WHO's step survey conducted in Tanzania revealed that 25.9% of Tanzanians were diagnosed with hypertension.^6^ The age-standardised prevalence of hypertension ranges between 19% and 25%.^7^ The prevalence is higher in urban regions (35%) than in rural areas (19%), with the highest burden being among adults.^8^

The most important modifiable contributor to the development of hypertension is an unhealthy lifestyle. Evidence points to a significant link between high blood pressure and heavy alcohol consumption, cigarette smoking, high sodium intake, physical inactivity, and poor dietary patterns.^9^ Heavy alcohol consumption promotes vasoconstriction, oxidative stress, and sympathetic activity.^10–13^ Tobacco smoking's effects on blood pressure are mediated by nicotine released in smoke. This is believed to promote sympathetic activity, vascular stiffness, and endothelial dysfunction in fields.^14–17^

Though the mechanism is not yet clear, high salt intake has been proven to cause extracellular fluid volume expansion in pressure natriuresis impaired and salt-sensitive persons, stimulating vascular resistance and central sympathetic activity^18–21^ On the other hand, physical activities and a diet rich in vegetables and fruits with low saturated and total fats create protective effects. The protective effect is created by attenuating vascular resistance, modulating sympathetic activity, widening the vascular lumen, promoting endothelial function, reducing oxidative stress, reducing body weight, and optimising metabolic and renal functions.^22–24^

Therefore, a change of lifestyle is a recommended key step for the prevention, management, and control of hypertension.^2,25,26^ WHO and the 7^th^ report of the Joint National Committee on Prevention, Detection, Evaluation, and Treatment of High Blood Pressure (JNC7) recommended cessation of cigarette smoking, moderation of alcohol consumption, moderate-intensity aerobic physical exercises, and healthy diets such as adoption of the DASH eating plan with reduced salt and saturated fats (and total fat) intake but increased vegetables and fruit consumption.^27,28^ These practices are more effective when combined than when adopted alone.^29^

The recommended diet plan together with physical exercise is likely to promote weight reduction which is also being endorsed as an approach to lower blood pressure.^1^ Weight reduction has demonstrated a significant impact on both systolic and diastolic blood pressure. Blood pressure was lowered by 1.6/1.1 mmHg for every kilogram of weight loss.^30^ In addition to the control of hypertension, the recommended lifestyle has benefits in preventing and controlling other cardiovascular and metabolic diseases.^31^

## METHODOLOGY

The study was an analytical cross-sectional study conducted from June to September 2020 at two referral hospitals in the Dodoma and Dar-es-Salaam regions. Dodoma is the capital city and centre of Tanzania, in eastern Africa. The city's population was 496,000 by 2020, and the prevalence of hypertension was 11.3%.^35^ Dar es Salaam is the largest city and industrial centre of Tanzania. The city's population is estimated to be 6,702,000 people by 2020, and the prevalence of hypertension was 24.3%.^35^

### Sample Size and Sampling

The study involved 311 randomly obtained hypertensive patients, equally allocated between the selected hospitals. The sample size was calculated using the Kish Leslie formula by plugging in a hypertension prevalence of 25.9%, a non-response rate of 5.3% obtained from the Tanzania STEPS survey^36^ and an error margin of 0.05. Only patients who received treatment for at least 6 months after a hypertension diagnosis and recorded more than three clinic visits were included. Hypertensive patients who were in critical conditions or with conditions that were likely to prejudice their responses, such as stroke and dementia, were excluded. The ethical clearance with reference number CB/229/308 was obtained from the University of Dodoma Institutional Research Review Committee, and permission was sought from the respective government and hospital authorities.

### Measurement of Variables

The sociodemographic information was obtained from the participants using a structured questionnaire. They included age, sex, marital status, level of education, health insurance coverage, and estimated monthly income. The dependent variable was adherence to lifestyle recommendations. It was broken down into specific recommended behaviours. Lifestyle behaviours were measured using a tool adapted from the WHO step survey of 2014.^6^ The nutrition show card was used to identify and describe the dietary lifestyle behaviours. Adherence to no tobacco smoking was defined as cessation or never smoking after a diagnosis of hypertension, as reported by participants. Adherence to alcohol moderation was defined as never consuming alcohol or consuming not more than 2 (1 for women) bottles or equivalent on any single day after a diagnosis of hypertension. Adherence to physical activity was defined as engaging in at least 30 minutes of daily moderate-to-vigorous aerobic physical activity for five days every week. Adherence to the recommended diet was defined as eating a diet rich in vegetables and fruits for at least 14 days a month.^6^ Adherence to each parameter was categorised as good if it met specific definition criteria and otherwise if it did not. The participant was considered overall adherent to the recommended lifestyle if they adhered to all four (4) recommended lifestyle modifications.

### Data Analysis

The data were entered and analysed using Statistical Product and Service Solution (SPSS), version 26 software. The factors associated with adherence to lifestyle recommendations were categorised and analysed by chi-square, followed by logistic regression for variables found to be significant. A level of significance (*P* value) was set to .05.

### Ethical Approval and Consent to Participate

Informed consent was obtained from all patients before collecting the data. Ethical approval for the study was obtained from the University of Dodoma.

## RESULTS

### Sociodemographic Characteristics of Participants

As summarised under Table 1, a total of 311 patients with hypertension responded to the interviewer-administered questionnaire. Among them, 58.8% were women, and 71.7% were married. Most participants (64.3%) were aged between 40 and 59 years, 53.4% had primary education, while 1% did not attend any formal education. 53.1% had health insurance coverage, 11.9% lived alone, and 46.3% had an average monthly income of less than 270,000 Tshs (which is equivalent to 116.88 USD). On the other hand, of all study participants, 47.9% had other comorbidities including diabetes (21.5%), asthma (10.0%), retinopathy (3.5%), heart failure (1.9%), kidney disorders (1.3%), neuropathy (1.3), and liver diseases (1.0%), and 7.7% had multiple comorbidities.

**TABLE 1:** Demographic and socio-economic Characteristics of Hypertensive Patients Attending Hypertension Clinics in Selected Hospitals in Tanzania (N=311)

Variables	n	%
Age group (years)		
18–39	35	11.3
40–59	200	64.3
60+	76	24.4
Sex		
Female	181	58.8
Male	130	41.2
Level of education		
Primary	166	53.4
Secondary	88	28.3
College	54	17.4
Non-formal	3	1.0
Participant income per month		
<270,000Tsh	144	46.3
270,001–520,000Tsh	81	26.0
520,001–760,000Tsh	70	22.5
760001–1000000Tsh	2	0.6
>1, 000,001Tsh	14	4.5
Health insurance coverage		
Yes	146	46.9
No	165	53.1
Live alone		
Yes	37	11.9
No	274	88.1

### Lifestyle Behaviours of Patients with Hypertension

As observed in Table 2, about 47.9% of surveyed patients had smoked tobacco at some point in their lifetime, and 1% were current smokers. 52.1% had ever consumed alcohol in their lifetime, 5.8% were current alcohol drinkers, and 37.9% were performing planned-specific exercises.

**TABLE 2:** Lifestyle Behaviours of Hypertensive Patients Attending Clinics in Selected Hospitals in Tanzania (N=311)

Variables	Variable category	n	%
**Lifestyle behaviours**			
Smoking			
Ever smoked any tobacco product	Yes	149	47.9
	No	162	52.1
Currently smoking	Yes	3	1.0
	No	308	99.0
Anyone in the household who smokes	Yes	21	6.8
	No	290	93.2
Anyone smokes at the workplace	Yes	4	1.9
	No	307	98.1
Alcohol consumption			
Ever consumed any alcohol	Yes	162	52.1
	No	149	47.9
Currently alcohol drinkers	Yes	18	5.8
	No	293	94.2
Frequency of drinking alcohol	1–3 days per month	4	22.2
	Less than a day	14	77.8
Physical activity			
Vigorous-intensity activity	Yes	72	23.2
	No	239	76.8
Moderate physical activity	Yes	310	99.7
	No	1	0.3
Specific planned physical activity	Yes	118	37.9
	No	193	62.1

### Adherence to Recommended Lifestyle among Hypertensive Patients

**FIGURE 1:**
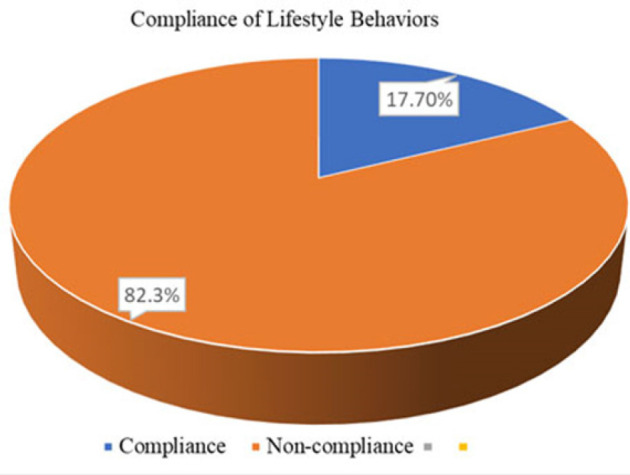
Adherence to Lifestyle Recommendations among Patients with Hypertension Attending Clinics in Selected Hospitals in Tanzania (N=311)

About 17.7% of the participants adhered to all four (4) lifestyle behaviours recommended for the treatment and control of hypertension. Adherence was higher for not smoking tobacco (99%) and moderate for alcohol consumption (94.2%). Adherence was lower to adequate fruit and vegetable intake (22.2%) and regular physical exercises (37.9%) (Figure 1).

### Factors Associated with Adherence to Recommended Lifestyle among Hypertensive Patients

As seen in Table 3, chi-square analysis revealed an association between age (*P=*.001) marital status (*P=*.044), occupation (*P*=.026) and knowledge of self-care practices (*P=*.036) with compliance with recommended lifestyle behaviors. When analysed further by logistic regression, participants who aged less than 60 years had 0.7 odds of being more adherent than their elder counterparts (OR= 0.74; 95% CI, 0.73 to 0.48; *P=*.001). Participants who were married had 0.51 odds of adhering compared to those who were unmarried (OR 0.74; 95% CI, 0.24 to1.06; *P=* .044]. Unemployed participants were almost 2 times more likely to adhere than the employed (OR 1.67; 95% CI, 0.92 to 3; *P=*.026). Hypertensive patients with adequate knowledge of self-care were almost two times more likely to adhere to the recommended lifestyle (OR=1.79, 95% CI, 1.09 to 2.87; *P=*.036). However, when odds were adjusted, only knowledge of self-care (aOR=2.32; 95% 95% CI, 1.082 to 3.471; *P=*.05) was statistically significant (Table 4).

**TABLE 3:** Factors Association of Compliance of Lifestyle behaviours of Hypertensive Patients Attending Clinics in Selected Hospitals in Tanzania

Variables	Compliance of Lifestyle Behaviours		Χ^2^	*P value*
	No n (%)	Yes n (%)		
Age group (years)				
18–39	33 (94.3)	3 (5.7)	7.527	0.023
40–59	196 (98.0)	4 (6.4)		
60+	72 (94.7)	4 (5.3)		
Marital status				
Union	178 (79.8)	7 (20.2)	3.369	0.044
Un-union	78 (88.6)	10 (11.4)		
Occupation				
Peasants	211 (82.1)	46 (17.9)	3.178	0.026
Employed	54 (100)	0 (0.0)		
Family history				
Yes	138 (83.6)	27 (16.4)	0.421	0.308
No	156 (94.5)	9 (5.5)		
Comorbidity				
Yes	126 (84.6)	23 (15.4)	1.045	0.191
No	129 (80.1)	32 (19.9)		
Knowledge				
Inadequate	79 (87.8)	11 (12.2)	2.596	0.036
Adequate	177 (80.1)	44 (19.9)		

**TABLE 4:** Association Between Socio-Demographic Factors and Adherence to Recommended Lifestyle by Logistic Regression Analysis

Variable	95% CI			*p-value*	95% CI			*p-value*
	COR	Lower	Upper		AOR	Lower	Upper	
Age group (years)								
<60	0.736	0.480	1.327	.308	0.625	0.303	1.288	.131
60+ Reference	1			.001				
Marital status								
union	0.507	O.243	1.058	.07	0.627	0.277	1.417	.262
Un-union (ref)	1			.001				
Occupation								
Unemployed	1.669	0.915	3.043	.095	0744	1.329	0.241	.744
Employed (ref)	1			.003				
Knowledge								
Reference	1			.000				
Adequate	1.785	1.085	2.870	.011	2.315	1.082	3.471	.05

## DISCUSSION

This study aimed to assess whether hypertensive patients adhered to the recommended lifestyle behaviours. Despite the advocacy for lifestyle modification among hypertensive patients, the burden of uncontrolled hypertension remained high. Even antihypertensive regimes remain insufficient to control hypertension without modifying risky lifestyles. Thus, this study found that there is an overall low adherence to the recommended lifestyle among hypertensive patients. Most of the participants complied with not smoking tobacco products and moderating alcohol consumption, but not eating the recommended diet or engaging in physical activities.

The overall low compliance with the recommended lifestyle among patients with hypertension has been reported in similar studies in Ethiopia and Nigeria.^33,34^ The studies used similar methods, and the nature of the participants involved was used in the current study. On the contrary, other studies conducted in Ghana, Nepal, and India reported higher adherence.^6,25,37^ The discrepancy in the findings could be due to differences in the quality of the tools used. The studies used structured questionnaires and participants aged over 30 years. These are people who are likely to have a higher perception of susceptibility to complications, socio-cultural factors. Also, the studies cited above are limited by the sample. For example, the sample size of the study conducted in Nepal used only 50 participants, while the study conducted in Saudi Arabia included only men.^38^

This study reported lesser involvement in physical activities and lower consumption of fruits and vegetables among hypertensive patients as compared to similar studies, which reported lower overall adherence.^39,40^ Lower engagement in regular physical activity could be due to work overload, lack of motivation, poor knowledge of the importance of physical activities, and limited physical abilities due to disease progression. Consumption of fruits and vegetables could have been affected by the availability of fruits and vegetables as well as knowledge of the importance of consumption of fruits and vegetables.

Despite lower overall adherence, there was high compliance with non-smoking and moderation or ceasing alcohol consumption. Similar findings were reported by other authors.^41^ A majority of the participants, who were previously smokers and/or alcohol consumers, halted after being diagnosed with hypertension. The cessation of alcohol consumption could have been fostered by the belief in the interaction between alcohol and antihypertensive drugs.^39^ Other patients could have avoided smoking and alcohol due to financial constraints implicated by disease limitations and the costs of purchasing antihypertensive drugs. However, the status of smoking and alcohol consumption behaviours could have been underreported by hypertensive patients.

This study found that adherence to recommended lifestyle modifications was significantly associated with age, marital status, employment status, and especially knowledge of self-care. Participants who were less than 60 years old were more likely to adhere than older patients. ^25^ This could be caused by limited ability to engage in physical activity and limited access to the recommended diet for reasons such as financial strain on the elderly. Also, the longer period after diagnosis could have made the elderly patient get used to the disease enough to ignore some of the recommended lifestyles.

Moreover, similar studies done in Ghana and Ethiopia ^25,37,38,41^ revealed that married hypertensive patients were more likely to adhere than patients who were not in marital union. This could be due to the support from the partner and motivation to adhere to the recommendations because of the sense of responsibility to prevent the potential complications of uncontrolled hypertension that could jeopardise their relationship, such as loss of sexual ability.

In this study, unemployed participants were more likely to adhere to the recommended lifestyle than employed participants. Self-employed patients may be more capable of controlling their daily schedules enough to include physical activities and determine their diet. Employed people are more likely to eat the diet they did not plan than unemployed people. Other unemployed patients could have been engaged in physically active economic activities while employed ones were doing sedentary jobs.

More significantly, patients who had adequate knowledge of self-care were almost two times more likely to adhere than patients without adequate knowledge, even after adjusting the odds ratios. This was supported by similar studies concluded elsewhere.^25,36, 37,40,41^ These patients could have been knowledgeable about hypertension, its complications, and how to prevent them. Therefore, they could have been more likely to comply with the recommended lifestyle modifications, although it should be noted that knowledge does not necessarily translate into practice.

## CONCLUSION AND RECOMMENDATIONS

Most hypertensive patients had poor adherence to the recommended lifestyle for the management of hypertension. Most patients adhered to avoiding tobacco smoking and moderating or ceasing alcohol consumption. A few of them ate the recommended diet and engaged in physical activities. More health interventions should be initiated to strengthen the healthcare system and public health programmes on lifestyle modifications, with more emphasis placed on diet modifications and physical activities. Patients who were aged less than 60 years, married, unemployed, and had adequate knowledge of self-care were more likely to adhere to the recommended lifestyle. Hypertensive patients should be encouraged and assisted to develop positive relationships from which they can get support. Also, other studies would explore the integration of lifestyle interventions with medication in the management of hypertension.

### Limitations

The study involved only hypertensive patients attending clinics at regional referral hospitals. The study could have missed important information from hypertensive patients who could not attend hypertension clinics or those who attended private hospitals. Also, recall bias could have affected the study findings. Moreover, this study did not assess adherence to the reduction in salt intake, which is an important recommendation for preventing and managing hypertension. Although it is not just any type of exercise that is considered beneficial to a person with hypertension, we didn't take into consideration the types of physical activities engaged by study participants. We could have done better if we assessed medication adherence together with lifestyle since both are essential in the management of hypertension.
